# A Case of Perityphlitic Abscess

**Published:** 1889-04

**Authors:** Eugene A. Smith

**Affiliations:** Assistant to Chair of Surgery, Medical Department, Niagara University; 66 High Street


### Clinical Reports.

### A CASE OF PERITYPHLITIC ABSCESS.

By EUGENE A. SMITH, M. D.,

Assistant to Chair of Surgery, Medical Department, Niagara University.

The following case occurred in the practice of Dr. James S. Smith, of Buffalo, and at his request I have written this report :

Dr. Smith was called, on the evening of February 2, 1889, to see H—— H——, a young man of medium size, eighteen years old, and a bookkeeper by occupation. The following history was obtained : The patient had always enjoyed good health, but he had for years been careless about defecation. His bowels at times moved daily, then again were not opened for two or even four days.

About nine o'clock, on February 2d, while at work, the patient began having "colic," as he expressed it, in his stomach. He went to a neighboring saloon and took a hot drink. From this he had temporary relief, but soon had to leave work and go home. In the afternoon, he vomited three or four times. Dr. Smith found him nauseated and suffering severely from spasmodic pain over the entire abdominal area. Morphine was given hypodermically, and hot applications ordered. His bowels had moved naturally during the morning, and also the day before. He had met with no injury, and his diet had not been unusual.

February 3d, the patient was found with a bounding pulse of 80; temperature, 99°. The pain was abated, and from this time on he suffered least when lying on his back, the pain being aggravated by lying on either side, and more so when lying on the left side. He vomited once after taking a cup of coffee. The tongue was clean, but in the succeeding few days it became furred. A milk diet was enjoined, and a powder of svapnia and bismuth given. An enema was followed by a small, costive movement.

February 4th, the pain became more localized, manifesting itself mainly in the right inguinal region. Pulse, 80; temperature, 103° F. The nausea was gone, and it returned no more. On February 5th, the pain was less marked, but tenderness was found, on pressure, in the right iliac region, just above Poupart's ligament. The abdomen was not tympanitic, and did not later become so. Temperature, 102½° F.; pulse, 80.

The next change in the patient's condition was noted on February 7th, when he complained of pain about the right knee-joint. An examination, however, revealed no local trouble. February 8th, this pain was accompanied by mild cramp of the muscles on the inner and posterior sides of the right thigh.

The treatment to this point had been expectant, svapnia and turpentine stupes being employed to quiet restlessness and soothe pain. Typhoid fever had early been excluded, and local inflammation about the head of the cecum diagnosticated. Abscess formation was now apprehended.

February 8th, an enema resulted only in a discoloration of the water, no fecal matter coming away. The next day, a slight dullness was found in the right iliac region, and on February 10th this was more marked over a spot, the size of a small saucer, just above the middle of Poupart's ligament. There was now constant pain just here, and extreme tenderness on pressure, which extended half-way to the free border of the ribs. No tumor presented, but this tenderness was abruptly contrasted with the normal condition of the abdomen above and on the left side. On this day, February 10th, the cramp of the muscles of the right thigh became so severe that relief was only obtained by anodynes, drawing up the right thigh, and resting the knee over a pillow. Temperature, 103° F.; pulse, 92. After an enema, containing glycerine ℥ii., a copious fecal movement relieved him greatly of pain, and his evening temperature was 102°; pulse, 92.

February 11th, Dr. Herman Mynter saw the patient, and agreed in the diagnosis arrived at by Dr. Smith and myself, of abscess about the head of the cecum. On the same afternoon, nine days from the onset of the trouble, the patient was anesthetized. Dr. Mynter then cut parallel to Poupart's ligament, about one-half inch above it and just external to its middle. Upon reaching the transversalis fascia, he inserted a hypodermic syringe-needle, which, being directed perpendicularly, touched the inner surface of the ileum without finding pus. Upon directing the needle upward, however, towards the kidney, an abscess cavity was found. He then made a free incision along the needle, upon which a foul, thick pus welled forth, filling two soup-plates, which together held about a pint and a half. The finger, inserted in the opening, found a large cavity, across which stretched a firm band of inflammatory formation. The cavity was thoroughly washed out with a 1-5000 corrosive sublimate solution, with the result of floating to the orifice a plug, seemingly a partly disintegrated blood-clot, having a strong fecal odor. A drainage-tube, three inches long, was sewed into the opening, and an antiseptic dressing applied. In the evening, he was resting well, with less pain, and a temperature of 101°.

The next day, February 12th, his temperature was 99½°; pulse, 76. He complained of soreness about the wound, but he felt pain in the thigh and heel of the right foot. This pain was explained, as were the earlier cramps and knee-pains, by irritation about the origin of the anterior crural and obturator nerves. No edema appeared, during the illness, in the limb of the affected side, but his first attempts at walking resulted in slight puffiness of the opposite leg and thigh.

February 13th, when the cavity was cleaned with a 1-5000 corrosive solution, a few teaspoonfuls of pus and a few blood-clots escaped, with a marked fecal odor. From this time, the patient steadily gained appetite and strength. February 16th, and again on February 22d, the wound was irrigated and redressed. On the former date, there was free oozing from granulations, and some fecal odor; on the latter date, the tube was withdrawn, there being no further fecal odor, and the cavity nearly filled. A dermatitis, excited by the corrosive compresses, raised the temperature to 99½° F. after the latter dressing. This was the only change in a regular temperature of 98½° F. from the day after the operation.

Since March 8th, the patient has been walking about, though still avoiding fatigue.

During his illness, the patient had no chills, but just after falling asleep he frequently broke out in a free perspiration.

A singular feature was observed during the twenty-four hours following each dressing in which the cavity was irrigated. The pulse-rate fell gradually till it reached 58. By the second day after the dressing, it again reached 72, and stayed so until the next dressing.

This was, doubtless, due to temporary inhibition through the pneumogastric, owing to interference with the accelerating function of the sympathetic ganglia. The gradual inhibition with the slow succeeding acceleration, point to a poisoning action on the abdominal sympathetic ganglia by the corrosive sublimate in the solutions.

66 High Street.

